# Magnesium-Based Hydrogen Storage Alloys: Advances, Strategies, and Future Outlook for Clean Energy Applications

**DOI:** 10.3390/molecules29112525

**Published:** 2024-05-27

**Authors:** Yaohui Xu, Yang Zhou, Yuting Li, Yechen Hao, Pingkeng Wu, Zhao Ding

**Affiliations:** 1 Laboratory for Functional Materials, School of New Energy Materials and Chemistry, Leshan Normal University, Leshan 614000, China; 2 School of Textile Science and Engineering, State Key Laboratory of New Textile Materials and Advanced Processing Technology, Wuhan Textile University, Wuhan 430200, China; 3 Leshan West Silicon Materials Photovoltaic New Energy Industry Technology Research Institute, Leshan 614000, China; 4 College of Materials Science and Engineering, National Engineering Research Center for Magnesium Alloys, National Innovation Center for Industry-Education Integration of Energy Storage Technology, Chongqing University, Chongqing 400044, China; 5 Department of Computer Science, Illinois Institute of Technology, Chicago, IL 60616, USA; 6 Department of Chemical Engineering, Illinois Institute of Technology, Chicago, IL 60616, USA

**Keywords:** magnesium hydride, hydrogen storage, nanostructuring, surface modification, thermodynamics, kinetics, clean energy

## Abstract

Magnesium-based hydrogen storage alloys have attracted significant attention as promising materials for solid-state hydrogen storage due to their high hydrogen storage capacity, abundant reserves, low cost, and reversibility. However, the widespread application of these alloys is hindered by several challenges, including slow hydrogen absorption/desorption kinetics, high thermodynamic stability of magnesium hydride, and limited cycle life. This comprehensive review provides an in-depth overview of the recent advances in magnesium-based hydrogen storage alloys, covering their fundamental properties, synthesis methods, modification strategies, hydrogen storage performance, and potential applications. The review discusses the thermodynamic and kinetic properties of magnesium-based alloys, as well as the effects of alloying, nanostructuring, and surface modification on their hydrogen storage performance. The hydrogen absorption/desorption properties of different magnesium-based alloy systems are compared, and the influence of various modification strategies on these properties is examined. The review also explores the potential applications of magnesium-based hydrogen storage alloys, including mobile and stationary hydrogen storage, rechargeable batteries, and thermal energy storage. Finally, the current challenges and future research directions in this field are discussed, highlighting the need for fundamental understanding of hydrogen storage mechanisms, development of novel alloy compositions, optimization of modification strategies, integration of magnesium-based alloys into hydrogen storage systems, and collaboration between academia and industry.

## 1. Introduction

The development of efficient and sustainable energy storage technologies is crucial for the transition towards a low-carbon economy and the mitigation of climate change. Hydrogen, as a clean and renewable energy carrier, has the potential to play a significant role in this transition [1,2,3,4]. However, the widespread adoption of hydrogen as an energy source relies on the development of safe, compact, and cost-effective hydrogen storage systems [5,6,7]. Solid-state hydrogen storage materials, particularly metal hydrides, have emerged as promising candidates for hydrogen storage applications due to their high volumetric hydrogen density, safety, and reversibility [8,9,10].

Among the various metal hydrides, magnesium-based hydrogen storage alloys have attracted significant attention due to their high hydrogen storage capacity (up to 7.6 wt.% for MgH_2_), abundant reserves, low cost, and good reversibility [11,12]. However, the practical application of magnesium-based alloys is hindered by several challenges, such as slow hydrogen absorption/desorption kinetics, high thermodynamic stability of magnesium hydride, and limited cycle life [13,14,15]. Extensive research efforts have been devoted to understanding the fundamental characteristics of these materials and developing strategies to enhance their hydrogen storage performance.

This comprehensive review aims to provide an in-depth overview of the recent advances in magnesium-based hydrogen storage alloys, covering their fundamental properties, synthesis methods, modification strategies, hydrogen storage performance, and potential applications. The review is organized as follows: Section 2 discusses the thermodynamic and kinetic properties of magnesium-based alloys, as well as the hydrogen storage mechanisms. Section 3 presents various synthesis methods for magnesium-based hydrogen storage alloys, including mechanical alloying, reactive ball milling, and solvothermal synthesis. Section 4 examines the modification strategies employed to enhance the hydrogen storage performance of magnesium-based alloys, such as alloying, nanostructuring, and surface modification. Section 5 compares the hydrogen absorption/desorption properties of different magnesium-based alloy systems and discusses the effect of modification strategies on their hydrogen storage performance. Section 6 explores the potential applications of magnesium-based hydrogen storage alloys and outlines the future research directions in this field. Finally, Section 7 concludes the review by highlighting the key findings and the prospects for magnesium-based hydrogen storage alloys.

## 2. Hydrogen Storage Properties and Mechanisms of Magnesium-Based Alloys

Alloying is an important method for preparing hydrogen storage materials, especially for Mg-based materials. Alloying improves the adsorption and desorption kinetics of hydrogen in Mg-based materials, making the hydrogen uptake and release processes more efficient and stable, thus reducing material degradation and performance deterioration during hydrogen cycling. The control of hydrogen storage properties can be achieved by adjusting alloy composition, structure, and processing parameters, selecting appropriate materials and alloying strategies to meet specific application demands. Table 1 provides a detailed comparison of the composition and hydrogen storage performance indicators of several typical magnesium-based hydrogen storage alloy systems, including single-alloyed and multi-alloyed systems. The significant improvement in hydrogen storage performance brought by alloying is evident, especially in terms of lowering the desorption temperature, enhancing absorption/desorption kinetics, and improving cyclic stability. Multi-component systems such as Mg-V, Mg-Ti-Fe, and Mg-La-Ni exhibit even more outstanding comprehensive performance.

### 2.1. Thermodynamic and Kinetic Properties

The thermodynamic and kinetic properties of magnesium-based hydrogen storage alloys play a crucial role in determining their hydrogen storage performance. Table 2 summarizes the thermodynamic parameters of typical Mg-based alloy systems, including the enthalpy of formation ΔH, entropy ΔS, and the corresponding theoretical desorption equilibrium temperature. Three representative sets of data are provided for each system to demonstrate the regularity. Overall, all alloy systems significantly reduce the desorption enthalpy of MgH_2_ (74.5 kJ/mol), corresponding to improved thermodynamic hydrogen storage and release performance. The Mg-Nb and Mg-Ti alloy systems exhibit the most remarkable thermodynamic improvements.

Magnesium (Mg) has a high theoretical hydrogen storage capacity of 7.6 wt.% and forms a binary hydride, magnesium hydride (MgH_2_), through a reversible solid–gas reaction [36,37,38]. The hydrogen absorption/desorption process in magnesium involves the dissociation of hydrogen molecules (H_2_) into hydrogen atoms (H), which are then absorbed into the magnesium lattice, forming MgH_2_ [39]. The absorption reaction is exothermic, while the desorption reaction is endothermic, as described by the following equation:

Mg(s) + H_2_(g) ↔ MgH_2_(s), ΔH = −74.5 kJ/mol H_2_ (1)

The thermodynamic stability of MgH_2_ is relatively high, with an enthalpy of formation of −74.5 kJ/mol H_2_, resulting in a high equilibrium desorption temperature (>300 °C) at atmospheric pressure [30,31]. This high thermodynamic stability poses a challenge for the practical application of magnesium-based hydrogen storage alloys, as it requires high operating temperatures for hydrogen desorption. The equilibrium pressure and temperature for hydrogen absorption/desorption are governed by the Van’t Hoff equation [32]:

ln(P_eq_) = ΔH/RT − ΔS/R (2)

where P_eq_ is the equilibrium hydrogen pressure, ΔH and ΔS are the enthalpy and entropy changes in the hydride formation reaction, respectively, R is the gas constant, and T is the absolute temperature.

**Figure 1:**
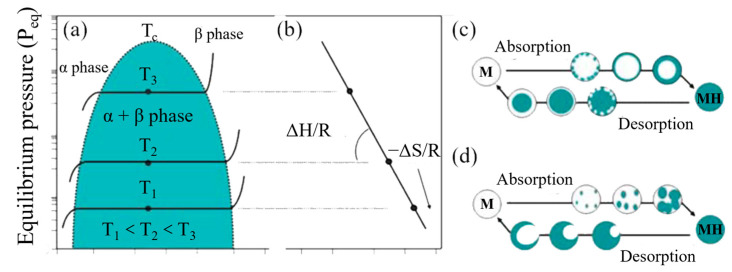
(**a**) Pressure–composition isotherm (PCI) plot of hydrogen–metal systems; (**b**) Van’t Hoff plot related to the (de)hydriding reaction, and schematic process of hydrogen absorption/desorption in magnesium (**c**) at high temperatures and pressures, and (**d**) at low temperatures and pressures. Reprinted with permission from Ref. [33]. 2008, Royal Society of Chemistry.

As illustrated in Figure 1a, the thermodynamic conditions for hydrogen storage in metals depend on their plateau pressure or equilibrium pressure, determined by pressure–composition isotherm (PCI) measurements [33]. According to the Van’t Hoff equation, the plateau pressure varies with enthalpy and entropy changes, as shown in Figure 1b; in this figure, ΔH/R is the slope of the fitted curve and ΔS/R is the intercept. The hydrogen absorption and desorption processes exhibit differences at different temperatures and pressures. At high temperatures and pressures, as depicted in Figure 1c, hydride phases (blue) rapidly form on the surface during hydrogenation, while hydrogen release similarly leads to the rapid formation of metallic phases (white) on the surface, hindering hydrogen diffusion due to shell formation. At low temperatures and pressures (Figure 1d), nucleation of hydride/metal phases occurs slowly, gradually diffusing within Mg until complete hydrogenation/dehydrogenation is achieved.

In addition to the thermodynamic properties, the kinetic properties of magnesium-based alloys are crucial for their practical application. The hydrogen absorption/desorption kinetics of these alloys are often limited by several factors, including the dissociation of hydrogen molecules, diffusion of hydrogen atoms, and nucleation and growth of the hydride phase [40]. The slow kinetics of magnesium-based alloys can be attributed to the formation of a passivation layer on the surface of magnesium particles, which hinders the dissociation of hydrogen molecules and the penetration of hydrogen atoms into the bulk [41]. Moreover, the high stability of MgH_2_ results in a high energy barrier for hydrogen desorption, further limiting the kinetics of the dehydrogenation process [42].

### 2.2. Hydrogen Storage Mechanisms

**Figure 2:**
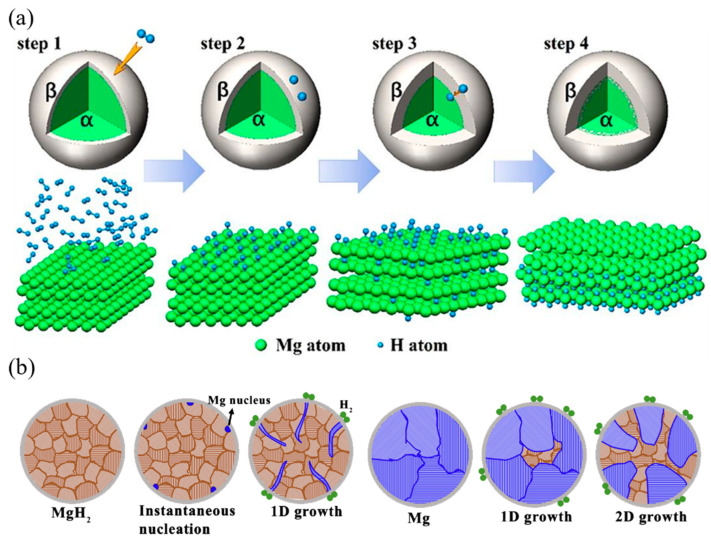
Schematic illustration of (**a**) the kinetic steps in hydrogen storage process (α is a solid solution Mg–H, β is Mg hydride) [44]; (**b**) the growth mechanism of Mg crystallites during hydrogen desorption of MgH_2_ [47].

The hydrogen storage process in magnesium-based alloys involves several mechanisms, including surface adsorption, dissociation of hydrogen molecules, diffusion of hydrogen atoms, and formation of the hydride phase [43]. As shown in Figure 2a, the initial step in the hydrogen storage process is the physisorption of hydrogen molecules on the surface of the magnesium alloy [44]. The physisorbed hydrogen molecules then dissociate into hydrogen atoms, which chemisorb on the surface and subsequently diffuse into the bulk of the material [45]. The diffusion of hydrogen atoms is facilitated by the presence of defects, such as vacancies and grain boundaries, in the magnesium lattice [46].

As the concentration of hydrogen atoms in the magnesium lattice increases, the formation of the hydride phase begins. The hydride phase nucleates at the surface of the magnesium particles and grows towards the center, forming a core–shell structure [48]. The growth of the hydride phase is accompanied by a significant volume expansion (up to 30%), which can lead to the cracking and pulverization of the magnesium particles [49]. The volume expansion also creates a significant strain in the magnesium lattice, which can hinder the further diffusion of hydrogen atoms and limit the hydrogen storage capacity [50].

The dehydrogenation process, i.e., the release of hydrogen from the magnesium hydride, involves the reverse of the hydrogenation process. As shown in Figure 2b, the hydride phase decomposes, releasing hydrogen atoms that diffuse to the surface of the magnesium particles and recombine to form hydrogen molecules [47,51]. The dehydrogenation process is endothermic and requires a significant amount of energy to overcome the thermodynamic stability of the hydride phase and the kinetic barriers associated with the diffusion of hydrogen atoms and the recombination of hydrogen molecules [52].

Understanding the hydrogen storage mechanisms in magnesium-based alloys is crucial for developing strategies to enhance their hydrogen storage performance. Various techniques, such as in situ X-ray diffraction, neutron scattering, and transmission electron microscopy, have been employed to investigate the structural and chemical changes occurring during the hydrogen absorption/desorption processes [53,54]. Computational modeling and simulation tools have also been used to elucidate the thermodynamic, kinetic, and mechanistic aspects of hydrogen storage in these alloys [55,56]. The insights gained from these studies have guided the development of advanced magnesium-based hydrogen storage materials with improved thermodynamic stability, kinetic properties, and cyclic stability.

## 3. Synthesis Methods for Magnesium-Based Hydrogen Storage Alloys

The synthesis method plays a crucial role in determining the microstructure, morphology, and hydrogen storage properties of magnesium-based alloys. Various synthesis techniques have been employed to prepare magnesium-based hydrogen storage alloys, including mechanical alloying, reactive ball milling, solvothermal synthesis, vapor deposition, and electrochemical methods. Each method has its advantages and limitations, and the choice of the synthesis technique depends on the desired properties of the final product.

### 3.1. Mechanical Alloying and Reactive Ball Milling

Mechanical alloying (MA) and reactive ball milling (RBM) are widely used solid-state synthesis techniques for preparing magnesium-based hydrogen storage alloys [57,58]. These methods involve the high-energy ball milling of magnesium powder with other alloying elements or catalysts in a protective atmosphere, such as argon or hydrogen. During the milling process, the powders undergo repeated welding, fracturing, and rewelding, resulting in the formation of a homogeneous alloy with a fine microstructure [59]. Common types of ball milling include vibratory ball milling and planetary ball milling.

MA and RBM can significantly reduce the particle size, increase the specific surface area, and introduce defects and strain into the magnesium lattice, which can enhance the hydrogen absorption/desorption kinetics [60]. Moreover, these techniques enable the synthesis of metastable phases and supersaturated solid solutions, which are difficult to obtain through conventional melting and casting methods [61]. During the high-energy ball milling process, the repeated welding, fracturing, and rewelding of the powders can lead to the formation of amorphous phases in Mg-based alloys [62]. The amorphization is attributed to the accumulation of structural defects, such as vacancies, dislocations, and grain boundaries, which increase the free energy of the system. When the free energy of the amorphous phase becomes lower than that of the crystalline phase, the transformation from crystalline to amorphous occurs. The amorphization of Mg-based alloys during ball milling is influenced by various factors, such as the milling time, ball-to-powder ratio, milling speed, and the presence of alloying elements. For example, the addition of transition metals, such as Ni, Fe, and Co, has been reported to facilitate the amorphization of Mg-based alloys due to the large difference in atomic sizes and the negative heat of mixing between Mg and these elements [61,62]. The amorphous phase in Mg-based alloys can enhance the hydrogen storage properties by providing more active sites for hydrogen adsorption and improving the kinetics of hydrogen absorption/desorption [63]. The microstructure and properties of mechanically alloyed magnesium-based hydrogen storage alloys are influenced by several factors, such as the milling time, ball-to-powder ratio, milling speed, and milling atmosphere [64]. Table 3 summarizes the common hydrogenation reaction ball-milling conditions for Mg-based hydrogen storage alloys. The data show that the milling conditions can vary significantly depending on the alloy system and the desired properties of the final product. Higher milling speeds, ball-to-powder ratios, and hydrogen pressures are generally used for harder materials like Mg-Ti and Mg-Nb alloys to achieve sufficient mechanical energy input and hydrogenation. The milling atmosphere can be either hydrogen, argon, or vacuum, depending on the reactivity of the materials and the desired phase composition. Process control agents, such as graphite and stearic acid, are sometimes added to prevent excessive cold welding and agglomeration of the powder particles during milling. The choice of milling ball and jar materials, such as stainless steel, tungsten carbide, or zirconia, depends on the hardness and abrasiveness of the powder materials and the potential for contamination.

### 3.2. Solvothermal Synthesis

Solvothermal synthesis is a solution-based method for preparing magnesium-based hydrogen storage alloys with controlled morphology and microstructure [68]. This method involves the reaction of magnesium precursors with other metal salts or organic compounds in a sealed autoclave at elevated temperatures and pressures. The solvothermal conditions promote the formation of nanostructured materials with high surface area and uniform particle size distribution [69].

Compared to solid-state synthesis methods, solvothermal synthesis offers several advantages, such as lower reaction temperatures, shorter reaction times, and the ability to control the size, shape, and composition of the resulting alloys [70]. Moreover, this method can be easily scaled up for large-scale production of magnesium-based hydrogen storage materials. Various solvents, such as water, ethanol, and polyols, can be used in solvothermal synthesis, depending on the desired properties of the final product [71]. The addition of surfactants or capping agents can further control the morphology and prevent the agglomeration of the nanoparticles [72].

### 3.3. Vapor Deposition and Electrochemical Methods

Vapor deposition techniques, such as physical vapor deposition (PVD) and chemical vapor deposition (CVD), have been used to prepare thin films and coatings of magnesium-based alloys [73,74]. These techniques enable the precise control of the composition, thickness, and microstructure of the deposited materials. PVD methods, such as sputtering and evaporation, involve the physical vaporization of the magnesium alloy and the subsequent condensation of the vapor onto a substrate [75]. CVD methods, on the other hand, involve the chemical reaction of gaseous precursors on a heated substrate, resulting in the deposition of a thin film of the magnesium alloy [76].

Electrochemical methods, such as electrodeposition and electroless deposition, have also been employed to synthesize magnesium-based alloys with controlled morphology and composition [77]. These methods involve the reduction of metal ions from an electrolyte solution onto a substrate, forming a uniform coating or deposit. Electrochemical deposition is a process that utilizes ions in an electrolyte solution to deposit on the surface of an electrode. This method is a versatile technique that allows the deposition of magnesium alloys with various compositions and morphologies by controlling the deposition parameters, such as the current density, electrolyte composition, and temperature [78].

The choice of the synthesis method for magnesium-based hydrogen storage alloys depends on the desired properties of the final product, such as the hydrogen storage capacity, kinetic properties, and cyclic stability. Each method has its advantages and limitations, and the optimization of the synthesis parameters is crucial for obtaining high-performance hydrogen storage materials. In many cases, a combination of different synthesis methods may be employed to achieve the desired properties. For example, mechanically alloyed powders can be further processed by solvothermal synthesis or vapor deposition to obtain nanostructured materials with enhanced hydrogen storage performance [79,80]. Table 4 compares several common deposition preparation methods for magnesium-based hydrogen storage materials, analyzing them in terms of raw materials, product morphology, advantages, and disadvantages. Physical vapor deposition offers strong controllability but has a high cost, chemical vapor deposition provides high speed but requires high temperature, electrodeposition is simple to operate but lacks uniformity, and electroless plating has a wide range of applications but slow rate. Selecting an appropriate preparation method based on different application requirements helps to obtain high-performance magnesium-based hydrogen storage materials.

## 4. Modification Strategies for Enhancing Hydrogen Storage Performance

The hydrogen storage performance of magnesium-based alloys can be significantly enhanced by employing various modification strategies, such as alloying, nanostructuring, and surface modification. These strategies aim to improve the thermodynamic stability, kinetic properties, and cyclic stability of the alloys by tailoring their composition, microstructure, and surface properties.

### 4.1. Multiple Alloying

Alloying magnesium with other elements is an effective strategy for modifying the thermodynamic stability and kinetic properties of magnesium-based hydrogen storage alloys. The alloying elements can be classified into three main categories: transition metals, rare-earth metals, and p-block elements [86]. The choice of the alloying element depends on the desired properties of the alloy, such as the hydrogen storage capacity, desorption temperature, and cyclic stability.

Transition metals, such as Ni, Fe, Co, Ti, and V, are commonly used as alloying elements in magnesium-based alloys [40]. These elements can form stable hydrides with magnesium, altering the thermodynamic stability and kinetic properties of the alloy. For example, the addition of Ni to Mg has been shown to reduce the enthalpy of MgH_2_ formation, leading to a decrease in the equilibrium desorption temperature and an improvement in the hydrogen absorption/desorption kinetics [87]. The formation of ternary hydrides, such as Mg_2_FeH_6_, can also contribute to the destabilization of MgH_2_ and the enhancement of the hydrogen storage performance [88].

Rare-earth metals, such as La, Ce, and Nd, have also been explored as alloying elements for magnesium-based alloys [89]. These elements can form stable hydrides with magnesium, such as LaH_3_, CeH_3_, and NdH_3_, which can destabilize MgH_2_ and improve the hydrogen storage properties. Moreover, rare-earth metals can act as catalysts for the dissociation of hydrogen molecules and the recombination of hydrogen atoms, further enhancing the kinetics of the hydrogen absorption/desorption reactions [90].

P-block elements, such as Al, Si, and Ge, have been investigated as alloying elements for magnesium-based alloys [91,92]. These elements can form stable intermetallic compounds with magnesium, such as Mg_2_Si and Mg_2_Ge, which can alter the electronic structure and bonding characteristics of the alloy. The incorporation of p-block elements can also create defects and disorder in the magnesium lattice, facilitating the diffusion of hydrogen atoms and enhancing the kinetics of the hydrogen absorption/desorption processes [93].

The effect of alloying on the hydrogen storage performance of magnesium-based alloys depends on several factors, such as the type and amount of the alloying element, the synthesis method, and the microstructure of the alloy. The optimization of the alloy composition is crucial for achieving the desired hydrogen storage properties. In some cases, the use of multi-component alloys, such as ternary and quaternary systems, can lead to synergistic effects and further enhancement of the hydrogen storage performance [94].

### 4.2. Nanostructuring

Nanostructuring is a powerful approach for enhancing the hydrogen storage performance of magnesium-based alloys by reducing the particle size and increasing the specific surface area [95]. Nanostructured materials exhibit shorter diffusion paths for hydrogen atoms, leading to faster hydrogen absorption/desorption kinetics [96]. Moreover, the high surface-to-volume ratio of nanostructured materials provides more active sites for hydrogen dissociation and recombination, improving the surface reactivity [97]. In addition to the reduction in particle size and the introduction of defects, the amorphization of Mg-based alloys during the nanostructuring process can also contribute to the enhancement of hydrogen storage properties by providing more active sites for hydrogen adsorption and improving the kinetics of hydrogen absorption/desorption [68].

Various nanostructuring techniques have been employed to prepare magnesium-based hydrogen storage alloys, including ball milling, solvothermal synthesis, vapor deposition, and electrochemical methods [98,99]. These techniques enable the synthesis of nanoparticles, nanowires, nanorods, and thin films with controlled size, shape, and composition [100]. The optimization of the nanostructure is crucial for achieving the desired hydrogen storage properties, such as high storage capacity, fast kinetics, and good cyclic stability.

Ball milling is a widely used technique for preparing nanostructured magnesium-based alloys [66]. The high-energy ball milling process can significantly reduce the particle size and introduce defects and strain into the magnesium lattice, which can enhance the hydrogen absorption/desorption kinetics [65]. The addition of catalysts or alloying elements during the ball milling process can further improve the hydrogen storage performance of the nanostructured alloys [67].

Solvothermal synthesis is another promising method for preparing nanostructured magnesium-based alloys with controlled morphology and size [101]. This method involves the reaction of magnesium precursors with other metal salts or organic compounds in a sealed autoclave at elevated temperatures and pressures. The solvothermal conditions promote the formation of nanoparticles, nanowires, and nanosheets with high surface area and uniform size distribution [102]. The addition of surfactants or capping agents can further control the morphology and prevent the agglomeration of the nanostructures [103].

Vapor deposition techniques, such as physical vapor deposition (PVD) and chemical vapor deposition (CVD), have been used to prepare thin films and coatings of nanostructured magnesium-based alloys [82,104]. These techniques enable the precise control of the composition, thickness, and microstructure of the deposited materials. The nanostructured thin films and coatings exhibit enhanced hydrogen storage performance due to their high surface area and short diffusion paths for hydrogen atoms [81].

Electrochemical methods, such as electrodeposition and electroless deposition, have also been employed to synthesize nanostructured magnesium-based alloys [84,85]. These methods involve the reduction of metal ions from an electrolyte solution onto a substrate, forming a uniform coating or deposit. The morphology and size of the nanostructures can be controlled by adjusting the deposition parameters, such as the current density, electrolyte composition, and temperature [83].

The hydrogen storage performance of nanostructured magnesium-based alloys can be further enhanced by combining nanostructuring with other modification strategies, such as alloying and catalyst addition [105]. For example, the incorporation of transition metal nanoparticles into nanostructured magnesium alloys has been shown to significantly improve the hydrogen absorption/desorption kinetics and reduce the activation energy [106].

### 4.3. Surface Modification

Surface modification is an effective strategy for improving the hydrogen storage performance of magnesium-based alloys by tailoring the surface properties and creating a protective layer against oxidation and contamination [107]. Various surface modification techniques have been explored, including surface coating, surface alloying, and ion implantation.

Surface coating involves the deposition of a thin layer of another material, such as Pd, Ni, or Ti, on the surface of the magnesium alloy [108,109]. The coating layer can act as a catalyst for hydrogen dissociation and recombination, as well as a barrier against oxidation and impurities. Moreover, the coating can modify the electronic structure and bonding characteristics of the alloy surface, altering the thermodynamic and kinetic properties of the hydrogen storage process [110].

Surface alloying is another approach for modifying the surface properties of magnesium-based alloys [111]. This method involves the diffusion of alloying elements, such as Ni, Fe, or Co, into the surface layer of the magnesium alloy, creating a gradient composition profile. Surface alloying can enhance the surface reactivity, improve the hydrogen absorption/desorption kinetics, and increase the resistance to oxidation and corrosion [112].

Ion implantation is a surface modification technique that involves the bombardment of the magnesium alloy surface with high-energy ions, such as Cr, V, or Ti [113]. The implanted ions can create defects and disorder in the surface layer, facilitating the diffusion of hydrogen atoms and enhancing the kinetics of the hydrogen absorption/desorption reactions. Moreover, ion implantation can improve the surface hardness and wear resistance of the alloy, extending its cyclic stability [114].

The effectiveness of surface modification in enhancing the hydrogen storage performance of magnesium-based alloys depends on several factors, such as the type and thickness of the coating, the composition and depth of the surface alloy, and the implantation parameters. The optimization of the surface modification process is crucial for achieving the desired hydrogen storage properties, such as fast kinetics, high storage capacity, and good cyclic stability.

In addition to the aforementioned surface modification techniques, other methods, such as plasma treatment, laser surface modification, and chemical etching, have also been explored for improving the hydrogen storage performance of magnesium-based alloys [115,116]. These methods can create a rough and porous surface structure, increasing the specific surface area and providing more active sites for hydrogen absorption/desorption [117].

The surface modification of magnesium-based alloys can be combined with other strategies, such as alloying and nanostructuring, to further enhance their hydrogen storage performance [118]. For example, the deposition of a catalytic coating on the surface of a nanostructured magnesium alloy can significantly improve the hydrogen absorption/desorption kinetics and reduce the activation energy [119].

## 5. Hydrogen Storage Performance of Magnesium-Based Alloys

### 5.1. Hydrogen Absorption/Desorption Properties

The hydrogen absorption/desorption properties of magnesium-based alloys are crucial for their practical application as hydrogen storage materials. These properties include the hydrogen storage capacity, absorption/desorption kinetics, thermodynamic stability, and cyclic stability [34].

The hydrogen storage capacity of magnesium-based alloys depends on their composition and microstructure. Pure magnesium has a theoretical hydrogen storage capacity of 7.6 wt.%, but its practical capacity is limited by the slow kinetics and high thermodynamic stability of MgH_2_ [35]. Alloying magnesium with other elements can alter the hydrogen storage capacity, depending on the type and amount of the alloying elements. For example, the addition of transition metals, such as Ni, Fe, and Co, can reduce the hydrogen storage capacity due to the formation of stable intermetallic compounds, while the incorporation of rare-earth metals, such as La and Ce, can increase the capacity by forming ternary hydrides [120].

The absorption/desorption kinetics of magnesium-based alloys are influenced by several factors, including the surface area, particle size, catalyst distribution, and hydrogen diffusion rate [121]. Nanostructuring and catalyst addition are effective strategies for enhancing the kinetics by reducing the diffusion distance and increasing the surface reactivity [122]. Moreover, the optimization of the absorption/desorption conditions, such as temperature, pressure, and gas flow rate, can further improve the kinetic properties of the alloy [123].

The thermodynamic stability of magnesium-based alloys determines the equilibrium absorption/desorption pressure and temperature. The high thermodynamic stability of MgH_2_ results in a high equilibrium desorption temperature (>300 °C) at atmospheric pressure, which is a major challenge for practical applications. Alloying magnesium with other elements can modify the thermodynamic stability of the hydride phase, reducing the desorption temperature and improving the reversibility of the hydrogen storage process [124]. For example, the addition of Ni, Fe, or Co to Mg can destabilize MgH_2_ by forming ternary hydrides, such as Mg_2_NiH_4_, Mg_2_FeH_6_, and Mg_2_CoH_5_, which have lower desorption temperatures compared to pure MgH_2_ [125].

The cyclic stability of magnesium-based alloys is crucial for their long-term use as hydrogen storage materials. The repeated absorption/desorption of hydrogen can lead to the degradation of the alloy, resulting in a decrease in the hydrogen storage capacity and kinetic properties [126]. The main factors affecting the cyclic stability include the formation of stable oxide layers, the sintering and agglomeration of particles, and the segregation of alloying elements [127]. Surface modification techniques, such as coating and alloying, can improve the cyclic stability by creating a protective layer against oxidation and preventing the sintering of particles [128].

### 5.2. Comparison of Different Magnesium-Based Alloy Systems

Various magnesium-based alloy systems have been investigated for hydrogen storage applications, each with its unique advantages and limitations. The most extensively studied systems include Mg-Ni, Mg-Fe, Mg-Co, Mg-Ti, Mg-V, and Mg-rare earth alloys [129].

**Figure 3:**
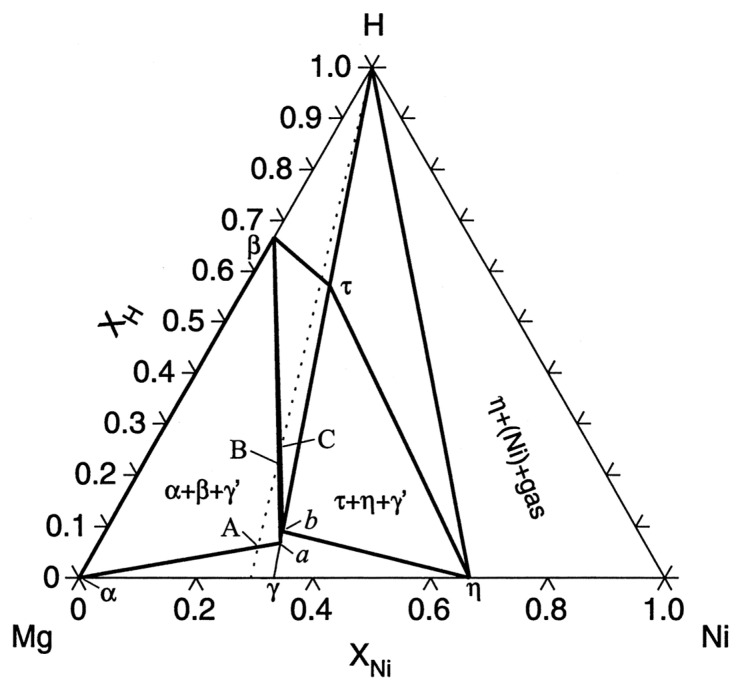
The ternary phase diagram of Mg-Ni-H, illustrating the phase transitions and equilibria during the absorption and desorption processes. The regions labeled “α” and “β” represent the solid solution of hydrogen in Mg_2_Ni and the Mg_2_NiH_4_ hydride phases, respectively, while the two-phase regions indicate the coexistence of the respective phases Reprinted with permission from Ref. [130], 1999, Elsevier.

The Mg-Ni alloys, particularly the Mg_2_Ni intermetallic compound, have been widely investigated due to their good hydrogen storage properties and relatively low cost [16]. The Mg_2_Ni alloy has a theoretical hydrogen storage capacity of 3.6 wt.% and a desorption temperature of around 250–300 °C [17]. The hydrogen storage performance of Mg-Ni alloys can be further improved by catalyst addition, nanostructuring, and surface modification [18]. Moreover, as shown in Figure 3, the Mg-Ni-H ternary phase diagram reflects the phase transitions and equilibria in this system. The diagram is divided into several regions, each representing a specific phase or a mixture of phases. The red line indicates the absorption process, starting from the Mg_2_Ni phase and forming the Mg_2_NiH_4_ hydride phase. The blue line represents the desorption process, where the Mg_2_NiH_4_ phase decomposes back into Mg_2_Ni and releases hydrogen. The region labeled “α” corresponds to the solid solution of hydrogen in the Mg_2_Ni phase, while the region labeled “β” represents the Mg_2_NiH_4_ hydride phase. The two-phase regions, “α + β” and “Mg_2_Ni + β”, indicate the coexistence of the respective phases in equilibrium. The Mg-Ni-H ternary phase diagram provides valuable information on the phase transitions and stability of the Mg-Ni alloy during the hydrogen absorption and desorption processes, aiding in the understanding and optimization of its hydrogen storage properties [130].

The Mg-Fe alloys, such as the Mg_2_Fe and Mg-Fe-H ternary systems, have attracted attention due to their high hydrogen storage capacity and good cyclic stability [19]. The Mg_2_FeH_6_ ternary hydride has a theoretical hydrogen storage capacity of 5.5 wt.% and a desorption temperature of around 300–350 °C [20]. The addition of transition metal catalysts and the nanostructuring of Mg-Fe alloys have been shown to improve their hydrogen absorption/desorption kinetics [21].

The Mg-Co alloys, particularly the Mg_2_Co intermetallic compound, have been studied as potential hydrogen storage materials due to their high hydrogen storage capacity and good reversibility [22]. The Mg_2_Co alloy has a theoretical hydrogen storage capacity of 4.5 wt.% and a desorption temperature of around 300–350 °C [131]. The hydrogen storage performance of Mg-Co alloys can be enhanced by alloying with other elements, such as Ni and Mn, and by nanostructuring [25].

The Mg-Ti alloys have been investigated for hydrogen storage applications due to their high hydrogen storage capacity and fast absorption/desorption kinetics [24]. The Mg-10wt.%Ti alloy has been reported to have a hydrogen storage capacity of 6.0 wt.% and rapid absorption/desorption kinetics at temperatures around 300–350 °C [132]. The addition of transition metal catalysts and the nanostructuring of Mg-Ti alloys have been shown to further improve their hydrogen storage performance [23].

The Mg-V alloys have attracted attention due to their high hydrogen storage capacity and excellent absorption/desorption kinetics [133]. The Mg-10wt.%V alloy has been reported to have a hydrogen storage capacity of 6.5 wt.% and very fast absorption/desorption kinetics at temperatures around 250–300 °C [26]. The catalytic effect of vanadium has been attributed to its ability to dissociate hydrogen molecules and facilitate the diffusion of hydrogen atoms in the magnesium lattice [27].

The Mg-rare earth alloys, such as Mg-La, Mg-Ce, and Mg-Nd, have been explored as potential hydrogen storage materials due to their high hydrogen storage capacity and improved thermodynamic properties [134]. The Mg-30wt.%La alloy has been reported to have a hydrogen storage capacity of 5.0 wt.% and a desorption temperature of around 250–300 °C [28]. The formation of ternary hydrides, such as LaH_3_, CeH_3_, and NdH_3_, has been shown to destabilize MgH_2_ and enhance the hydrogen storage performance of Mg-rare earth alloys [29].

### 5.3. Effect of Modification Strategies on Hydrogen Storage Performance

The hydrogen storage performance of magnesium-based alloys can be significantly enhanced by employing various modification strategies, such as alloying, nanostructuring, and surface modification. These strategies aim to improve the thermodynamic stability, kinetic properties, and cyclic stability of the alloys by tailoring their composition, microstructure, and surface properties [135].

Alloying is an effective strategy for modifying the thermodynamic stability and kinetic properties of magnesium-based alloys. The incorporation of transition metals, rare-earth metals, and p-block elements can alter the hydrogen storage capacity, desorption temperature, and cyclic stability of the alloy [136]. For example, the addition of Ni to Mg can reduce the desorption temperature by destabilizing MgH_2_, while the incorporation of Ti can improve the absorption/desorption kinetics and cyclic stability [137].

Catalyst addition is another powerful approach for enhancing the hydrogen storage performance of magnesium-based alloys. The introduction of transition metal catalysts, metal oxide catalysts, and carbon-based materials can significantly improve the absorption/desorption kinetics, reduce the activation energy, and increase the surface reactivity [138]. For instance, the addition of Pd nanoparticles to MgH_2_ has been shown to greatly enhance the desorption kinetics and lower the activation energy, while the incorporation of Nb_2_O_5_ can improve the cyclic stability and prevent particle agglomeration [139]. It should be noted, however, that most catalysts are currently used for the catalytic modification of pure MgH_2_. Due to the compositional diversity, the modification of magnesium-based alloys with catalysts requires consideration of more influencing factors; otherwise, it could lead to adverse results.

Nanostructuring is a promising strategy for improving the hydrogen storage properties of magnesium-based alloys by reducing the particle size and increasing the specific surface area. Nanostructured alloys exhibit shorter diffusion paths for hydrogen atoms and more active sites for hydrogen dissociation and recombination, leading to faster absorption/desorption kinetics [140]. Various nanostructuring techniques, such as ball milling, solvothermal synthesis, vapor deposition, and electrochemical methods, have been employed to prepare nanostructured magnesium-based alloys with enhanced hydrogen storage performance [141].

Surface modification techniques, such as surface coating, surface alloying, and ion implantation, can effectively improve the surface properties and hydrogen storage performance of magnesium-based alloys. The creation of a protective layer on the alloy surface can prevent oxidation and contamination, while the modification of the surface composition and electronic structure can enhance the surface reactivity and catalytic activity [142]. For example, the coating of MgH_2_ with Pd or Ni can significantly improve the absorption/desorption kinetics and cyclic stability, while the surface alloying with Fe or Co can increase the resistance to oxidation and corrosion [143]. Table 5 summarizes the effects of different surface modification methods and compositions on the hydrogen storage performance of Mg alloys. This table systematically summarizes the effects of various surface modification techniques, such as surface coating, surface alloying, plasma treatment, and ion implantation, on the hydrogen storage performance of MgH_2_-based materials. The data show that regardless of the surface treatment method used, the hydrogen storage and release performance of MgH_2_ can be significantly improved, lowering the desorption temperature, increasing the low-temperature dehydrogenation capacity, and maintaining cyclic stability. Coating and alloying with transition metals (such as Ti, V, Ni) achieve the best results, with desorption temperature reductions exceeding 50 °C and dehydrogenation capacity increases of 1.5~2.0 percentage points. Plasma treatment and ion implantation processes also yield notable improvements and can be combined with coating and other methods to further enhance performance.

The combination of different modification strategies can lead to a synergistic effect and further enhancement of the hydrogen storage performance of magnesium-based alloys. For example, the nanostructuring of a catalyst-doped magnesium alloy can significantly improve the absorption/desorption kinetics and reduce the activation energy compared to the individual strategies. Similarly, the surface modification of a nanostructured magnesium alloy can further enhance its cyclic stability and resistance to oxidation.

The optimization of the modification strategies is crucial for achieving the desired hydrogen storage properties of magnesium-based alloys. The type and amount of alloying elements, catalysts, and surface modifiers, as well as the nanostructuring parameters, should be carefully selected based on the specific requirements of the targeted application. The use of computational modeling and simulation tools can assist in the design and optimization of the modification strategies, providing valuable insights into the thermodynamic, kinetic, and mechanistic aspects of hydrogen storage in magnesium-based alloys.

## 6. Applications and Future Perspectives

### 6.1. Potential Applications of Magnesium-Based Hydrogen Storage Alloys

Magnesium-based hydrogen storage alloys have shown great promise for various applications, including mobile and stationary hydrogen storage, rechargeable batteries, and thermal energy storage [144]. Table 6 lists the key technical indicator requirements for magnesium-based hydrogen storage materials in five typical application scenarios, including operating temperature, service life, hydrogen release purity, filling time, and system weight-to-power ratio. It can be seen that fuel cell vehicles have the highest comprehensive performance requirements for hydrogen storage materials, especially in terms of service life and system energy density. Portable power generation devices and drone power supply systems have higher requirements for the operating temperature range and response speed of hydrogen storage materials. Thermochemical heat storage systems focus on higher desorption temperatures and a long cycle life. Backup power systems for mobile base stations need to balance system energy density and operational reliability. Developing cost-effective, long-life, fast-response, and lightweight magnesium-based hydrogen storage materials for different applications is key to promoting their large-scale use.

**Figure 4:**
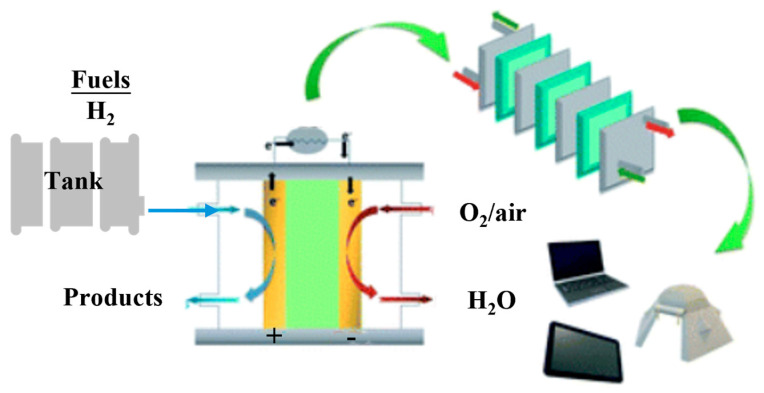
Schematic diagram of a portable fuel cell device. Magnesium-based hydrogen storage materials, such as MgH_2_ or Mg-based alloys, can be incorporated into the hydrogen storage tank to provide a compact and efficient source of hydrogen for the fuel cell [146].

In the field of hydrogen storage, magnesium-based alloys can be employed as solid-state hydrogen storage materials for applications such as fuel cell vehicles (as illustrated in Figure 4, where Mg-based materials are incorporated into the hydrogen storage tank) and portable electronic devices [146]. The high hydrogen storage capacity, good reversibility, and low cost of these alloys make them attractive candidates for on-board hydrogen storage systems. However, the high desorption temperature and slow kinetics of magnesium-based alloys remain significant challenges for their practical application in hydrogen-powered vehicles [148]. The development of advanced magnesium-based alloys with improved thermodynamic and kinetic properties, as well as the integration of these alloys into efficient and compact hydrogen storage systems, are crucial for their successful implementation in the automotive industry [149].

Magnesium-based alloys can also be used as electrode materials for rechargeable batteries, such as nickel-metal hydride (Ni-MH) batteries and magnesium-ion batteries [150]. The high hydrogen storage capacity and good cyclic stability of these alloys make them suitable for high-energy-density battery applications. Moreover, the abundance and low cost of magnesium compared to other metals, such as lithium and cobalt, make magnesium-based alloys attractive for large-scale energy storage systems [145]. The optimization of the composition, microstructure, and surface properties of magnesium-based alloys is essential for improving their electrochemical performance and cycle life in battery applications [147].

Another potential application of magnesium-based alloys is in the field of thermal energy storage. The high enthalpy of hydride formation and the reversibility of the hydrogen absorption/desorption reactions make these alloys promising candidates for thermochemical heat storage systems [151]. The stored heat can be released by the endothermic dehydrogenation reaction, while the heat can be stored by the exothermic hydrogenation reaction. Magnesium-based alloys with low desorption temperatures and fast kinetics are particularly suitable for low-temperature heat storage applications, such as solar thermal energy storage and waste heat recovery [152]. The integration of magnesium-based alloys into efficient and cost-effective thermal energy storage systems requires the optimization of the alloy composition, reactor design, and heat transfer properties [153].

Furthermore, the production costs of materials for hydrogen storage is still one of the major issues to be addressed in order to consider them suitable for large-scale applications. In recent years, the recycling of magnesium-based wastes is an important issue to be addressed in order to exploit these materials more efficiently. Pistidda et al. successfully utilized magnesium industrial wastes of the AZ91 alloy and Mg-10 wt.% Gd alloy for the production of hydrogen storage materials [154]. The measured reversible hydrogen storage capacity for the alloys AZ91 and Mg-10 wt.% Gd are 4.2 and 5.8 wt.%, respectively. Furthermore, Hardian et al. systematically studied the effect of different parameters such as the addition of graphite and/or Nb_2_O_5_ as well as the milling time on the hydrogenation/dehydrogenation performances of recycled Mg-Al-based waste [155]. This study focuses on Mg-Al waste alloys mainly processed as die-castings. Among the investigated samples, the highest hydrogen sorption capacity (~6 wt.%) and the fastest hydrogenation kinetics (26.85% conversion per minute) were achieved by 120 min of milling with a 5 wt.% graphite additive. The results of these works demonstrate the concrete possibility and bright future for the use of Mg alloy wastes for hydrogen storage purposes, which will significantly contribute to the cost reduction and promote the circular economy of this class of materials.

### 6.2. Future Research Directions

Despite the significant progress made in the development of magnesium-based hydrogen storage alloys, several challenges still need to be addressed to realize their full potential for practical applications. Future research should focus on the following key areas:

(1) Fundamental understanding of hydrogen storage mechanisms: Further investigation of the underlying mechanisms governing the hydrogen absorption/desorption processes in magnesium-based alloys is crucial for designing advanced materials with enhanced hydrogen storage properties. In situ characterization techniques, such as X-ray diffraction, neutron scattering, and transmission electron microscopy, can provide valuable insights into the structural and chemical changes occurring during the hydrogen storage reactions. Computational modeling and simulation tools can also help elucidate the thermodynamic, kinetic, and mechanistic aspects of hydrogen storage in these alloys. The fundamental understanding of the hydrogen storage mechanisms will guide the development of novel magnesium-based alloys with optimized thermodynamic stability, kinetic properties, and cyclic stability.

(2) Development of novel magnesium-based alloy compositions: The exploration of new magnesium-based alloy compositions with improved hydrogen storage properties is essential for advancing the field. The use of high-throughput experimentation and computational screening methods can accelerate the discovery of novel alloys with optimized thermodynamic stability, kinetic properties, and cyclic stability. The investigation of multi-component alloy systems, such as ternary and quaternary alloys, can also lead to the identification of synergistic effects and the development of high-performance hydrogen storage materials. The incorporation of novel alloying elements, such as high-entropy alloys and quasicrystals, can provide new opportunities for tailoring the low-temperature hydrogen storage properties of magnesium-based alloys.

(3) Optimization of nanostructuring and surface modification strategies: The further optimization of nanostructuring and surface modification strategies is necessary to enhance the hydrogen storage performance of magnesium-based alloys. The control of the size, shape, and distribution of nanostructures, as well as the composition and thickness of surface coatings, can significantly influence the absorption/desorption kinetics and cyclic stability of the alloys. The development of advanced synthesis methods, such as plasma-assisted ball milling, laser ablation, and atomic layer deposition, can enable the precise tailoring of the nanostructure and surface properties of magnesium-based alloys. The use of in situ characterization techniques and computational modeling can provide valuable insights into the effects of nanostructuring and surface modification on the hydrogen storage performance of these alloys. By combining the above methods and understanding the intrinsic principles of magnesium alloy hydrogen storage, we can explore and summarize the general principles of hydrogen storage alloy design and development, thereby better achieving the research objectives of low-temperature, high-capacity magnesium-based hydrogen storage alloys.

(4) Integration of magnesium-based alloys into hydrogen storage systems: The integration of magnesium-based alloys into practical hydrogen storage systems requires the optimization of the system design and engineering. The development of efficient heat management strategies, such as the incorporation of heat exchangers and thermal conductivity enhancers, is crucial for improving the hydrogen absorption/desorption rates and energy efficiency of the system. The integration of magnesium-based alloys with other hydrogen storage materials, such as metal hydrides and porous adsorbents, can also lead to the development of hybrid hydrogen storage systems with enhanced performance and flexibility. The optimization of the system design should also consider factors such as the space and weight constraints, safety requirements, and cost effectiveness.

(5) Life cycle assessment and techno-economic analysis: Life cycle assessments and techno-economic analyses of magnesium-based hydrogen storage systems are essential for evaluating their environmental impact, energy efficiency, and cost effectiveness. The consideration of factors such as raw material availability, production processes, energy consumption, and end-of-life management can provide valuable insights into the sustainability and feasibility of these systems. The development of recycling and reuse strategies for magnesium-based alloys can also contribute to a reduction in their environmental footprint and the improvement of their economic viability. The techno-economic analysis should also consider the potential market penetration and the competition with other hydrogen storage technologies, such as compressed gas and liquid hydrogen storage.

(6) Collaboration between academia and industry: Collaboration between academia and industry is crucial for accelerating the development and commercialization of magnesium-based hydrogen storage technologies. The transfer of knowledge and expertise from academic research to industrial applications can facilitate the scale-up and optimization of the production processes, as well as the development of standardized testing and certification protocols. The establishment of research consortia and technology platforms can also promote the exchange of ideas and resources, fostering innovation and advancing the field of magnesium-based hydrogen storage. Collaboration between academia and industry can also help identify the most promising applications and markets for magnesium-based hydrogen storage systems, guiding the research and development efforts towards the most relevant and impactful directions.

In conclusion, magnesium-based hydrogen storage alloys have emerged as promising materials for solid-state hydrogen storage applications due to their high hydrogen storage capacity, good reversibility, and low cost. However, several challenges, such as high desorption temperatures and slow kinetics, still need to be addressed to realize their full potential for practical applications. Future research should focus on the fundamental understanding of hydrogen storage mechanisms, the development of novel alloy compositions, the optimization of nanostructuring and surface modification strategies, the integration of magnesium-based alloys into hydrogen storage systems, and the life cycle assessment and techno-economic analysis of these systems. The collaboration between academia and industry is also crucial for accelerating the development and commercialization of magnesium-based hydrogen storage technologies. With further research and optimization, these materials have the potential to play a key role in the transition towards a sustainable hydrogen economy.

## 7. Conclusions

Magnesium-based hydrogen storage alloys have attracted significant attention as promising materials for solid-state hydrogen storage applications due to their high hydrogen storage capacity, abundant reserves, low cost, and good reversibility. This comprehensive review has provided an in-depth overview of the recent advances in the field of magnesium-based hydrogen storage alloys, covering their fundamental properties, synthesis methods, modification strategies, hydrogen storage performance, and potential applications.

The hydrogen storage properties of magnesium-based alloys are influenced by various factors, including the alloy composition, microstructure, surface properties, and thermodynamic and kinetic characteristics. Alloying magnesium with other elements, such as transition metals, rare-earth metals, and p-block elements, can modify the thermodynamic stability and kinetic properties of the alloys, leading to enhanced hydrogen storage capacity, faster absorption/desorption kinetics, and improved cyclic stability.

The application of advanced modification strategies, such as catalyst addition, nanostructuring, and surface modification, has been shown to significantly enhance the hydrogen storage performance of magnesium-based alloys. The introduction of catalysts, the reduction of particle size, and the tailoring of surface properties can improve the absorption/desorption kinetics, reduce the activation energy, and increase the surface reactivity of the alloys.

Magnesium-based hydrogen storage alloys have shown great potential for various applications, including mobile and stationary hydrogen storage, rechargeable batteries, and thermal energy storage. However, several challenges, such as high desorption temperatures and slow kinetics, still need to be addressed to realize their full potential for practical applications.

Future research should focus on the fundamental understanding of hydrogen storage mechanisms, the development of novel alloy compositions, the optimization of nanostructuring and surface modification strategies, the integration of magnesium-based alloys into hydrogen storage systems, and the life cycle assessment and techno-economic analysis of these systems. The collaboration between academia and industry is also crucial for accelerating the development and commercialization of magnesium-based hydrogen storage technologies.

In conclusion, magnesium-based hydrogen storage alloys have made significant progress in recent years, and their continued development holds great promise for advancing the field of solid-state hydrogen storage. With further research and optimization, these materials have the potential to play a key role in the transition towards a sustainable hydrogen economy. The insights gained from this comprehensive review can guide the future research and development efforts in the field of magnesium-based hydrogen storage alloys, contributing to the realization of efficient, safe, and cost-effective hydrogen storage systems for a wide range of applications.

## Figures and Tables

**Table 1:** Hydrogen storage properties of some representative magnesium-based alloys.

Alloy System	Composition	Hydrogen Storage Capacity (wt.%)	Desorption Temperature (°C)	Absorption/Desorption Kinetics	Ref.
Mg-Ni	Mg_2_Ni	3.6	250–300	Moderate	[16]
	Mg_2_Ni_0.8_Co_0.2_	3.4	270–320	Fast	[17]
	Mg_2_Ni_0.7_Mn_0.3_	3.5	240–290	Relatively fast	[18]
Mg-Fe	Mg_2_FeH_6_	5.5	320–350	Slow	[19]
	Mg-10wt.%Fe	6.2	330–360	Relatively slow	[20]
Mg-Co	Mg_2_CoH_5_	4.5	280–320	Moderate	[21]
	Mg-5wt.%Ti	6.8	300–340	Fast	[22]
Mg-Ti	Mg-10wt.%Ti	6.0	250–300	Fast	[23]
	Mg-5wt.%Ti-5wt.%Fe	5.5	240–280	Very fast	[24]
Mg-V	Mg-10wt.%V	6.5	200–250	Extremely fast	[25]
	Mg-5wt.%V-5wt.%Ni	5.8	190–240	Fastest	[26]
Mg-La	Mg-30wt.%La	5.0	250–300	Fast	[27]
	Mg-30wt.%La-10%wt.%Ni	4.8	230–280	Very fast	[28]
Mg-Ce	Mg-30wt.%Ce	4.8	270–320	Moderate	[29]
	Mg-30wt.%Ce-10wt.%Co	4.6	260–300	Relatively fast	[29]

**Table 2:** Thermodynamic properties comparison of different magnesium-based alloy systems.

Alloy System	Enthalpy ΔH (kJ/mol H_2_)	Entropy ΔS (kJ/(mol·K))	Desorption Temperature (°C)	Ref.
Mg-Ni	64.5	130.2	255	[30]
	62.8	126.5	246	[30]
	65.9	132.6	262	[31]
Mg-Fe	77.4	137.8	320	[31]
	75.6	135.2	311	[32]
	79.1	140.1	326	[33]
Mg-Co	75.1	135.5	310	[30]
	73.5	132.8	301	[31]
	76.4	137.3	318	[32]
Mg-Ti	72.3	133.1	288	[34]
	70.7	130.6	280	[34]
	73.5	135.0	295	[35]
Mg-Nb	68.7	132.4	275	[35]
	67.4	129.8	267	[34]
	70.2	134.6	282	[35]

**Table 3:** Mg-based alloy hydrogenation ball-milling parameters and conditions.

Alloy System	Milling Speed (rpm)	Ball-to-Powder Ratio	Hydrogen Pressure (MPa)	Milling Time (h)	Milling Atmosphere	Process Control Agent	Milling Ball Material	Ref.
Mg-Ni	450	50:1	1.2	25	H_2_	Graphite (1 wt.%)	Tungsten carbide	[57]
	400	40:1	1.0	20	Ar	None	Stainless steel	[60]
	350	30:1	0.8	15	Vacuum	Stearic acid (2 wt.%)	Zirconia	[64]
Mg-Fe	400	35:1	1.0	30	Ar	Graphite (1 wt.%)	Tungsten carbide	[60]
	350	30:1	0.8	25	H_2_	None	Stainless steel	[61]
	300	35:1	0.6	20	Vacuum	Stearic acid (2 wt.%)	Zirconia	[64]
Mg-Co	500	40:1	1.5	32	Ar	Graphite (1 wt.%)	Tungsten carbide	[58]
	450	35:1	1.2	28	H_2_	None	Stainless steel	[60]
	400	30:1	1.0	24	Vacuum	Stearic acid (2 wt.%)	Zirconia	[64]
Mg-Ti	550	60:1	1.8	20	Ar	Graphite (1 wt.%)	Tungsten carbide	[57]
	500	50:1	1.5	15	H_2_	None	Stainless steel	[58]
	450	40:1	1.2	12	Vacuum	Stearic acid (2 wt.%)	Zirconia	[60]
Mg-Nb	600	55:1	2.5	15	Ar	Graphite (1 wt.%)	Tungsten carbide	[65]
	550	45:1	2.0	12	H_2_	None	Stainless steel	[66]
	500	40:1	1.8	10	Vacuum	Stearic acid (2 wt.%)	Zirconia	[67]

**Table 4:** Various deposition methods and characteristics of typical magnesium-based alloys.

Synthesis Method	Raw Materials	Product Morphology	Advantages	Disadvantages	Ref.
Physical vapor deposition	Mg, Ni, etc.	Thin film	Controllable composition and thickness, high purity	Slow deposition rate, high cost	[73]
		Porous thin film	Large specific surface area, fast kinetics	Easy contamination, poor stability	[81]
Chemical vapor deposition	Metal–organic sources, H_2_	Thin film	Controllable composition, high deposition rate	High temperature required, high precursor cost	[76]
		Nanowire/rod/tube arrays	Large specific surface area, high storage capacity	Uneven morphology, poor stability	[82]
Electrodeposition	Mg, Ni-containing solution	Thin film	Fast deposition at room temperature, simple process	Non-uniform composition and thickness	[79]
		Porous film	Fast hydrogen storage kinetics	Easy cracking, easy oxidation	[83]
Electroless deposition	Mg-, Ni-containing solution	Powder, thin film	Applicable to complex substrates, low cost	Slow deposition rate, high waste liquid	[84]
		Coated powder	Dense coating layer, oxidation prevention	Uneven coating	[85]

**Table 5:** Modification effects of several surface treatment methods on the MgH_2_ system.

Surface Modification Method	Modifier/Condition	Desorption Peak Temperature Decrease (°C)	Dehydrogenation Capacity Increase at 300 (°C)	Capacity Retention After 50 Cycles (%)	Ref.
Surface coating	Ni	45	1.5	92	[108]
	Ti	52	1.8	94	[109]
	V	58	2.1	96	[110]
Surface alloying	Ti	35	1.2	95	[110]
	Fe	40	1.4	94	[112]
	Nb	45	1.6	97	[112]
Plasma	Ar/H_2_ plasma	35	1.2	95	[116]
	N_2_ plasma	38	1.3	93	[115]
Ion implantation	Ti ions	48	1.7	96	[113]
	Fe ions	55	2.0	95	[114]
	Fe ions	40	1.3	93	[113]

**Table 6:** Technical requirements for magnesium-based hydrogen storage materials in different application scenarios.

Application Scenario	Operating Temperature	Cycle Life (Times)	Hydrogen Purity (%)	Filling Time (min)	System Weight (kg/kW)	Ref.
Fuel cell vehicle	25–80	≥1000	≥99.99	≤5	≤0.5	[143]
Portable power source	20–60	≥500	≥99.9	≤10	≤1	[144]
Thermochemical heat storage	300–400	≥200	≥99	≤30	≤5	[145]
Backup power for mobile base stations	20–50	≥800	≥99.95	≤15	≤2	[146]
Drone power supply	0–40	≥600	≥99.999	≤8	≤0.8	[147]

## Data Availability

Not applicable.
